# Development of a Method to Potentially Substitute Direct Evaluation of Mesopic Visual Acuity in Drivers

**DOI:** 10.3390/ijerph18094733

**Published:** 2021-04-29

**Authors:** Marta Garcia-Rojo, Cristina Bonnin-Arias, Eva Chamorro, Cristina Alvarez-Peregrina, Celia Sanchez-Ramos

**Affiliations:** 1 Optometry and Vision Department, Facultad de Óptica y Optometría, Avda, Arcos de Jalón 118, 28037 Madrid, Spain; marta.g.rojo@gmail.com (M.G.-R.); cbonnina@ucm.es (C.B.-A.); celiasr@opt.ucm.es (C.S.-R.); 2 Neuro-Computing and Neuro-Robotics Research Group, Universidad Complutense de Madrid, Avda, Arcos de Jalón 118, 28037 Madrid, Spain; evachamorrogutierrez@gmail.com; 3 Faculty of Biomedical and Health Sciences, Universidad Europea de Madrid, 28670 Madrid, Spain

**Keywords:** mesopic visual acuity, photopic visual acuity, contrast, night driving, aging, driver behavior, vision

## Abstract

(1) Background: In mesopic lighting conditions, or under adverse environmental circumstances, visual information is reduced, which increases the risk of traffic accidents. This effect could be reduced with a precise evaluation of the visual function under mesopic conditions, but it is difficult to replicate in clinics. This study aims to develop an easy-to-adopt method to evaluate mesopic visual acuity (VA) in drivers. (2) Methods: Prospective and observational study in drivers. logMAR mesopic VA was compared with photopic VA measured under different combinations of contrast charts and filters to find the combination that responds best to mesopic conditions. (3) Results: Fifty-six drivers were examined. The best correlation was found with an 80% density filter and a Weber contrast chart of 20%. The logMAR VA for this combination was 0.01 ± 0.11, which was close to the mesopic VA values (0.01 ± 0.12). The difference between both logMAR VA was 0.00 ± 0.06 (R = 0.86; *p* ≤ 0.001; ICC = 0.86). (4) Conclusions: The use of 20% contrast optotypes and the interposition of an 80% filter under photopic conditions provide VA values similar to those measured under mesopic lighting conditions, making this simple system a good predictor of mesopic VA values.

## 1. Introduction

Driving is a complex and eminently visual task. It depends on the combination of three agents: the driver, the environment, and the vehicle. Therefore, the three main factors to consider in road safety are the driver’s functional state, the type and state of the vehicle, and the condition of the driving routes. It is also important to highlight that driving is the preferred mode of transportation for young and older people [1].

The constant rise in the number of drivers and the use of road transportation has caused an increase in the road accident rate. In 2015, the World Health Organization (WHO) considered road accident rates to be a public health problem worldwide [2]. It is estimated that that every year, between 1,200,000 and 1,500,000 people die in traffic accidents, and between 20 and 50 million suffer some type of trauma related to driving [3].

Vision directly affects driving performance and, as a result, road safety. Alterations of the visual function, such as the loss of visual acuity (VA), the decrease in the visual field, and some ocular pathologies, have been associated with an increase in the risk of suffering traffic accidents [4]. Furthermore, this risk is higher in mesopic or night illumination conditions (0.01 to 3 cd/m^2^) than in photopic or daylight illumination conditions (>3 cd/m^2^) [3,5]. The risk is also higher in the elderly than in young people [6].

Human vision in mesopic environments presents some particularities, such as a reduction in color vision, a decrease in the ability to discriminate details, and a shift in the light efficiency towards middle wavelengths. In these mesopic conditions, both photoreceptors are involved with different degrees of participation. However, in photopic conditions, visual perception is characterized by good discrimination of color vision and details. Cone photoreceptors are responsible for this discrimination, because they provide high spatial resolution and properly process the color vision [7]. So, there are more difficulties in detecting low-contrast or poorly illuminated objects during night driving, which implies longer reaction and braking times and longer stopping distances [8,9].

Because of aging in the neural and optical processes, older people suffer more severe visual perception losses in night vision than in daylight vision [5,6,10]. Moreover, because of their decrease in self-confidence, due to a reduction in the visual, cognitive, and functional abilities related to aging, many older drivers limit their driving to certain situations [5]. In this sense, it is known that the physiological changes in the visual system related to aging may affect the attitude and the tendency to suffer accidents [11].

A well-known factor contributing to the deficiency of the mesopic visual function in all drivers is night myopia, mainly characterized by the increase in the defocus under conditions of low illumination. This is caused by a change in the refractive properties of the eye when the pupil is dilated (mydriasis), since the peripheral area of the optical system focuses less effectively than the central area (spherical aberration). According to Mehra et al., a myopic effect of one diopter is produced under the illumination conditions of 10^−2^ cd/m^2^, and myopia increases up to two diopters for illumination levels around 3·10^−5^ cd/m^2^ [12]. Several studies have tried to establish the influence of night myopia in driving performance. Cohen’s remarkable work analyzed the visual function of professional drivers, showing a positive correlation between night myopia over −0.75 D and the accident rate during night driving [13]. The main conclusion of the above-mentioned work was the suggestion of performing a test to measure night myopia in all drivers, with particular attention on professional drivers.

Since, under mesopic illumination or poor weather conditions, visual information is reduced, contour discrimination gets worse, and there is a substantial loss of color perception, a proper assessment of vision could decrease the risk of suffering night driving accidents. Regarding this, the evaluation of VA in night driving conditions must be a priority in the normalization and regularization of the visual exploration of drivers. Photopic VA alone is not a good predictor of night driving ability, and mesopic VA seems relevant for night driving [14]. Due to the small number of studies evaluating predictors for night driving ability, further research is needed.

A luminance adaptation of the exam room could be considered to assess visual function in mesopic conditions. However, this would require a complex normalization and calibration process that would be hard to introduce in medical evaluation centers. We suggest evaluating photopic VA with a low contrast test and filters of different optical densities, aiming to predict the mesopic VA.

The main objective of the present work is to develop an easy to implant method to evaluate mesopic VA by determining which mixture of contrasts of the optotype chart and filters in photopic vision correlates best with the values of the mesopic VA, avoiding the difficult task of changing the environmental illumination during the test.

## 2. Materials and Methods

A prospective, observational and cross-sectional study in drivers was carried out. VA in several conditions was measured by an optometrist in a laboratory, where environmental light conditions could be modified. All the subjects wore the correct refractive correction.

Early Treatment Diabetic Retinopathy Study (ETDRS) charts were used to determine VA with different Weber contrasts (100%, 20%, 10%, 5%, 2.5% and 1.25%) in the Precision Vision Illuminator Cabinet™ backlit cabin, which provides uniform illumination of 170 cd/m^2^ [15]. VA was expressed in the logMAR scale, so lower values correspond to better VA.

**Figure 1:**
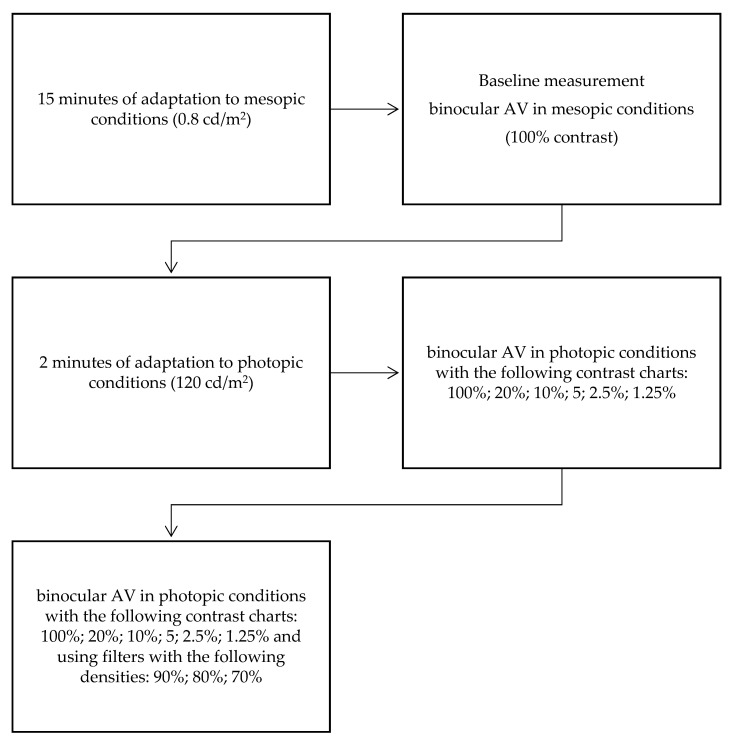
Flowchart of the visual examination steps.

Figure 1 shows a flowchart with the different steps of the visual examination. The first exam was conducted in the laboratory where mesopic conditions were identical (0.8 cd/m^2^), and after dark-light adaptation for 15 min. The optometrist took the high contrast binocular VA using ETDRS charts with 100% contrast. This VA was the reference to achieve without changes in environmental light conditions, so it was the baseline measure.

After two minutes of light adaptation, VA in photopic conditions (120 cd/m^2^) with different filters was taken. Filters were used in the following order:

- Without any filter;
- With neutral density filters of 90% decreasing density (10% transmittance);
- With neutral density filters of 80% decreasing density (20% transmittance);
- With neutral density filters of 70% decreasing density (30% transmittance).

The same procedure was followed with the different Weber contrast charts (20%, 10%, 5%, 2.5% and 1.25%).

In line with the International Organization for Standardization (ISO) 8596:2010 regulations concerning the ratio of luminance between the optotype and the background, a neutral optical density filter was used to simulate mesopic conditions. The filter used was a Rosco E-Color 211 (Rosco-Iberian SA, Madrid, Spain) of 13.7% transmittance, which provides an optotype luminance of 5 cd/m^2^. The filter was placed between the optotype and the luminous background. The Mavolux 5032B USB luxmeter (Mavo Monitor USB, Gossen, Nürnberg, Germany) was used for the photometric verification of the environment and the optotype chart.

The statistical analysis was performed using the software Statgraphics Centurion XVI (Statgraphics Technologies, Inc., The Plains, VA, USA) and SPSS Statistics (SPSS Inc., Chicago, IL, USA). A descriptive statistic of the variables evaluated was carried out and the assumption of normality was checked using the Shapiro–Wilk test.

Differences between photopic and baseline (mesopic) measurements were calculated to assess which measures in photopic conditions better simulate mesopic VA. To compare these measures, the Pearson correlation coefficient (R) and the *p*-value were calculated. In addition, the intraclass correlation coefficient (ICC) was applied to evaluate the association of these measures. The scale proposed by Landis and Koch to assess the quality of the ICC is as follows: 0 = poor, 0.01–0.20 = mild, 0.21–0.40 = moderate, 0.61–0.80 = substantial and 0.81–1.00 = almost perfect (Kramer and Feinstein, 1981).

In addition, the relation of the quantitative variables was analyzed using a Bland–Altman (B&A) type chart. The B&A plot analysis is a simple way to evaluate a bias between the mean differences and to estimate an agreement interval, within which 95% of the differences of the second method, compared to the first one. Data can be analyzed both as a plot of unit differences and as a plot of percentage differences [16].

The size of the sample was calculated from the data of a pilot study in which photopic VA was evaluated in 30 people. The acceptable sampling error was a value of 0.2 and a confidence level of 0.95. Given the possibility of losses, the sample size was increased by 10%. The result of the pilot study indicated that the sample size should be 50 people.

The current study was conducted according to the guidelines of the Declaration of Helsinki, and approved by the Ethics Committee of “Hospital Clínico San Carlos” (protocol code C.I. 12/366-E).

## 3. Results

The sample consisted of 56 subjects, between 20 and 71 years of age (44.01 ± 18.42). Regarding gender, 37.50% were males and 62.50% were females. A total of three people were excluded because of their ocular pathologies: age-related macular degeneration (AMD) and retinal detachment. They did not have a valid driver’s license. They were distributed into two groups: under 50 (n = 30; age 28.70 ± 5.28) and over 50 years old (n = 26; age 63.46 ± 7.57). Table 1 shows the demographic and refractive situation of the participants.

There was a statistically significant decrease (*p* ≤ 0.001) in the maximum contrast of mesopic VA (0.01 ± 0.12) compared to the maximum contrast photopic VA (−0.06 ± 0.06). The decrease in VA caused by the reduction in the level of ambient lighting was also significant in both age groups; 0.11 ± 0.14 for the group over 50 (*p* ≤ 0.001), and −0.03 ± 0.06 for the group under 50 (*p* = 0.003). Table 2 shows the results with the different contrast and illumination conditions.

**Figure 2:**
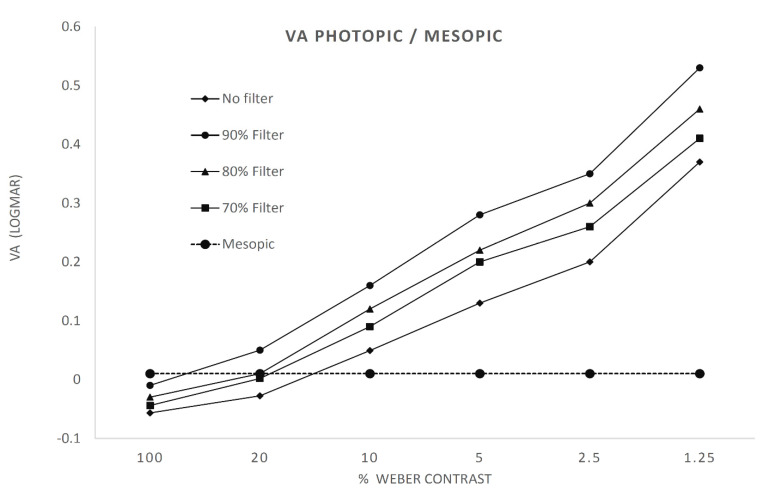
Correlation between mesopic and photopic visual acuity measured with different contrasts.

Regarding the comparison of mesopic VA in the lab conditions vs. VA with different filters and contrast in photopic conditions, the best correlation was found with the interposition of an 80% density filter with a Weber contrast test of 20%, as Figure 2 shows that the logMAR VA for this combination was 0.01 ± 0.11, which was close to the mesopic VA value (0.01 ± 0.12). The difference between both logMAR VA was 0.00 ± 0.06 (R = 0.86; *p* ≤ 0.001; ICC = 0.86). Table 3 shows the results of all the combinations made in the total sample and in the different age groups.

**Figure 3:**
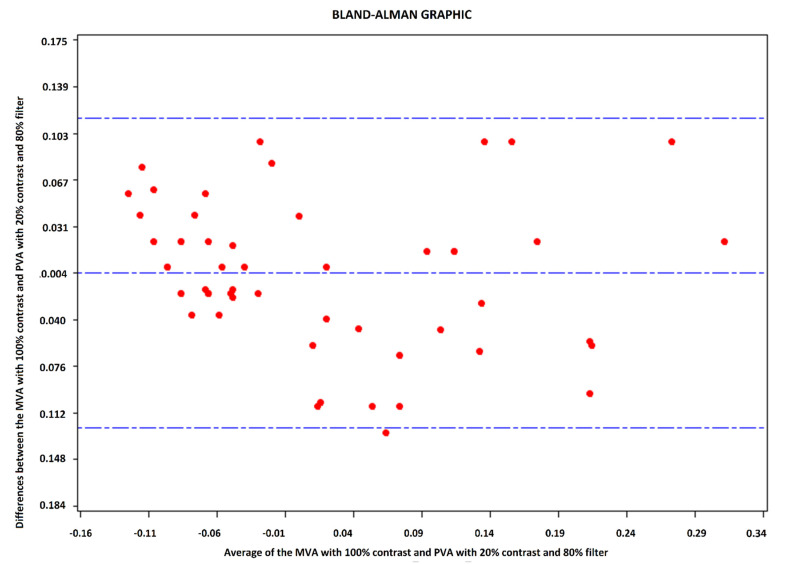
Bland–Altman plot of differences between the mesopic VA with 100% contrast and photopic VA with 20% contrast and a density filter of 80% absorption.

A B&A plot representing the difference of the means divided by the average of both means is used to analyze the relationship between the quantitative variables. A difference of zero between the two means implies that both results are similar in value. In our case, the resulting mean was −0.004, so the mean value of both methods is very close. Figure 3 shows that they were linked because there were no extreme points on the figure.

## 4. Discussion

Road safety depends on the user’s functional status, vehicle, and road condition [17]. However, the driver makes the final decisions, so he/she is responsible for the proper development of driving. Driving is a complex task that requires perception functions and motor skills. Namely, vision is the most important source of perceptual information for the driver [1]. Driving in a state in which the required skills are endangered may cause injuries to oneself and others [6,17].

The European Working Group for Vision explicitly concludes that the most important visual function parameters in order of relevance for safe driving are VA, contrast sensitivity, visual field, and sensitivity to glare [18]. Different national regulations have unified the minimum requirements of visual function necessary to grant driver’s licenses. However, the process is incomplete, because while carrying out visual exams in mesopic conditions is recommended in order to get a driver’s license, specific restrictive thresholds are not specified. In practice, photopic VA values are still the only reference regarding the state of vision in most countries.

The demographic data suggest a rapid increase in the number of people over 65, a group which is becoming a growing portion of the population. For instance, in 30 years, the number of over 65s in the USA will double, going from 40.2 million in 2010 to 81.2 million in 2040 [19]. The improvements in lifestyle, the higher life expectancy, and the increase in physical activity in this age group imply an increase in the number of elderly people driving; a demographic that will continue to grow. Therefore, it is necessary to encourage older people to keep their visual health in good condition.

The natural aging process causes a reduction in physical and cognitive abilities [6,20]. Specifically, there are gradual changes due to both the optic-ocular system and neuronal factors that affect the visual function [17]. Some studies have already found that older drivers have diminished VA, low contrast sensitivity, and are also influenced more by glare and halo [21].

In our study, for all the conditions evaluated, we also observed significantly lower VA values in the older subjects compared to the younger ones.

The loss of VA is more pronounced in older drivers as the lens becomes yellower and less transparent and the pupil becomes smaller [9,22,23,24], so drivers with cataracts have a worse driving performance than drivers without cataracts [25]. Many authors report that VA is stable until age 50, with a gradual decrease from 51 years onward [9,26]. For this reason, we divided our sample into those over and under 50.

Namely, differences of 30% in mesopic VA of 100% contrast, and between 15 and 41% in photopic VA with different contrast, were found. These values agree with those obtained by Wood in 2014 and Gruber in 2013 [6,14]. This is relevant because in the group of participants over 50, who due to their age show a decrease in photopic VA, there is also an additional deterioration of visual quality in low light conditions. That means that they can have regular VA values during daytime driving and significantly lower values during night driving.

Older subjects presented a more pronounced decrease in VA due to lighting conditions. This decrease was 8% in the younger subjects and 24% in the subjects over 50. These results are similar to those obtained by Sivack in 1981, who carried out one of the first studies relating mesopic VA and age. The study explored the readability of motorway signals in a real driving environment at night. The results show that the readability distance in older participants was between 23% and 35% higher than that of the younger group [27].

In this work, we used, as a baseline method, the measurements of VA in mesopic conditions, which were achieved by controlling the light in a special laboratory. Other baseline methods using existing tests, such as Nyktometer or Mesotest, could be used, but they are focused on contrast sensitivity instead of visual acuity [28,29]. In our baseline conditions, we recorded mesopic VA values of 0.01 ± 0.12 for the total of the sample, −0.06 ± 0.06 for people under 50, and 0.09 ± 0.11 for people over 50. Thus, we observed significantly lower values of mesopic VA in the older group. These data are similar to those published in 1998 by Mehra [12]. In Mehra’s study, the VA values oscillated between 0.3 and 0.8 depending on the level of mesopic illumination (0.045–0.12 cd/m^2^). Puell’s work, carried out in 2004, provides similar values [9]. Therefore, our study reinforces the idea that VA evaluation in different illumination and contrast conditions is essential to test the aptitudes required to obtain the driver’s license, especially for older drivers.

Regarding the illumination conditions, it is known that VA increases with luminance. Wood et al. [14] showed that for luminance values around 5 cd/m^2^ (mesopic limit), VA remained practically constant and very low. As the level of illumination increases, VA progressively improves, until it reaches a maximum and saturates for values around 100 cd/m^2^ (photopic illumination). In that study, the room conditions were modified to respond to both mesopic and photopic conditions, with a luminance of 0.8 cd/m^2^ and 120 cd/m^2^ respectively.

The relationship between VA at different levels of illumination and depending on age was already published in 1966 and 1995 by Burg and Frisen [30,31]. All of them found a higher loss of vision in people over 60 years of age. On the other hand, the pioneer studies of Sivack and Sturgis show the relationship between visual degradation and night driving [27,32]. Other researchers have investigated the interplay of these three variables: VA, illumination, and age. For instance, Forbes found a significant reduction in VA with decreasing light conditions, as well as with increasing age, concluding that the continuous decrease in VA is more significant as the environmental lighting approaches scotopic levels [33].

Traditionally, the determination of static VA has been used as a measure of the ability of the visual system to solve objects in well-lit environments. In most countries, static VA determination tests are established as a predictor of visual function status and are used as a restrictive value in the examination that allows access to the driver’s license. Only lately has the evaluation of the driver’s mesopic VA been added to the medical examination tests for driving licenses. This recommendation is still being studied by the authorities of various countries [6].

Nowadays, the measurement of mesopic VA implies the purchase of special tests or instruments or even adequate room lighting. One of the instruments used for the mesopic VA measure is Mesotest^®^ II (Oculus, Wetzlar, Germany), a standardized unit approved by the German Ophthalmological Society. It has been widely studied for testing mesopic vision and glare sensitivity [34,35,36,37]. Our study proposes a simple and easy-to-adopt method for the measure of mesopic VA.

Different levels of luminance in a test room can be achieved by neutral filters of different optical density. The luminance of the test seen through a filter can be calculated according to the expression L = L_0_ × T.

Among the few experimental studies on night driving, the one by Allen stands out [38]. In that paper, the author studied how as the illumination of the retina decreases due to the interposition of monochromatic lenses, the distance at which objects are detected on the road also decreases. There is a significant decrease in the night detection distance with a transmittance of around 70%. Thus, the effect on the detection of obstacles, caused by reductions in road lighting was highlighted.

Before undertaking our study, we calculated that if L were mesopic luminance (0.8 cd/m^2^) and L_0_ were photopic luminance (120 cd/m^2^), the result transmittance (T) would be 0.007. This would imply a filter transmission percentage of 0.7% and an absorption of 99.3%. The theoretical filter obtained to simulate mesopic lighting conditions would be an excessively dark filter. For this reason, in this work, we sought the combination of filters of different optical densities (90%, 80%, and 70%) with a VA test of several contrasts (100%, 20%, 10%, 5%, 2.5% and 1.25%) to find the best mesopic predictor. However, it would be interesting for further studies to compare the results in mesopic-induced conditions with exams in real-world driving conditions and study the effect of eye pathologies in the proposed test.

## 5. Conclusions

It is important to evaluate the mesopic VA in people that seek to obtain a driver’s license. This study proposes a practical and economical method that is also easy to implement in examination centers. The use of 20% contrast optotypes and the interposition of an 80% filter under photopic conditions provide VA values similar to those measured under mesopic lighting conditions, making this system a good predictor of mesopic VA values.

## 6. Patents

Sanchez-Ramos, C.; Bonnin-Arias, C. (2018). Spain. Patent N°. ES2634365 (B1). Madrid, Spain: Oficina Española de Patentes y Marcas.

## Figures and Tables

**Table 1:** Demographic and refractive situation of the subjects.

Item	Younger	Older	Total Sample
	(Aged 20–50)	(Aged 51–80)	
Age (years) (mean ± SD)	29.8 ± 4.4	62.3 ± 4.3	46.1 ± 17.0
Gender			
Male	16.67%	61.54%	37.50%
Female	83.33%	38.46%	73.21%
Refractive error (D)			
Emmetropes	56.67%	53.85%	55.36%
Myopes	33.33%	23.08%	28.57%
Hyperopes	10.00%	23.08%	16.07%

**Table 2:** Visual Acuity of the total sample and by ages in different lighting conditions and with different contrast.

Lighting Conditions	Contrast	VA (Mean ± SD)				
		Total Sample	Younger	Older	Differences	*p*
Mesopic	100%	0.01 ± 0.12	−0.06 ± 0.06	0.09 ± 0.12	−0.15 ± 0.09	0.0001
Photopic	100%	−0.06 ± 0.06	−0.09 ± 0.01	−0.02 ± 0.07	−0.07 ± 0.04	0.0001
Photopic	20%	−0.03 ± 0.08	−0.08 ± 0.02	0.02 ± 0.09	−0.10 ± 0.07	0.0001
Photopic	10%	0.04 ± 0.11	−0.03 ± 0.05	0.13 ± 0.12	−0.15 ± 0.09	0.0001
Photopic	5%	0.13 ± 0.15	0.04 ± 0.09	0.23 ± 0.14	−0.18 ± 0.12	0.0001
Photopic	2.5%	0.20 ± 0.15	0.10 ± 0.08	0.31 ± 0.13	−0.21 ± 0.11	0.0001
Photopic	1.25%	0.37 ± 0.12	0.25 ± 0.09	0.50 ± 0.15	−0.25 ± 0.12	0.0001

**Table 3:** Visual Acuity of the total sample and by age group with the different filters and contrast.

Filter	Contrast	VA in Photopic Environment (Mean ± SD)				
		Total Sample	Younger	Older	Differences	*p*
90%	100%	−0.01 ± 0.10	−0.06 ± 0.04	0.04 ± 0.11	−0.11 ± 0.08	0.0001
	20%	0.05 ± 0.13	−0.02 ± 0.07	0.14 ± 0.14	−0.16 ± 0.11	0.0001
	10%	0.16 ± 0.13	0.09 ± 0.08	0.25 ± 0.13	−0.17 ± 0.11	0.0001
	5%	0.28 ± 0.15	0.20 ± 0.08	0.37 ± 0.15	−0.17 ± 0.12	0.0001
	2.5%	0.35 ± 0.15	0.27 ± 0.08	0.44 ± 0.16	−0.18 ± 0.12	0.0001
	1.25%	0.53 ± 0.17	0.43 ± 0.09	0.64 ± 0.20	−0.20 ± 0.13	0.0001
80%	100%	−0.03 ± 0.08	−0.08 ± 0.04	0.01 ± 0.10	−0.09 ± 0.07	0.0001
	20%	0.01 ± 0.11	−0.06 ± 0.04	0.08 ± 0.11	−0.14 ± 0.08	0.0001
	10%	0.12 ± 0.13	0.06 ± 0.07	0.20 ± 0.13	−0.14 ± 0.10	0.0001
	5%	0.22 ± 0.15	0.13 ± 0.07	0.33 ± 0.14	−0.20 ± 0.11	0.0001
	2.5%	0.29 ± 0.16	0.19 ± 0.08	0.41 ± 0.16	−0.22 ± 0.13	0.0001
	1.25%	0.46 ± 0.17	0.36 ± 0.07	0.58 ± 0.17	−0.23 ± 0.12	0.0001
70%	100%	−0.04 ± 0.07	−0.09 ± 0.02	0.00 ± 0.08	−0.09 ± 0.06	0.0001
	20%	0.00 ± 0.10	−0.07 ± 0.03	0.07 ± 0.11	−0.13 ± 0.08	0.0001
	10%	0.09 ± 0.13	0.02 ± 0.06	0.17 ± 0.14	−0.15 ± 0.11	0.0001
	5%	0.20 ± 0.15	0.11 ± 0.09	0.30 ± 0.15	−0.19 ± 0.12	0.0001
	2.5%	0.26 ± 0.16	0.18 ± 0.08	0.36 ± 0.17	−0.19 ± 0.13	0.0001
	1.25%	0.41 ± 0.17	0.31 ± 0.08	0.52 ± 0.19	−0.20 ± 0.14	0.0001
